# Downregulation of GSDMB by EBV-encoded miR-BART12-3p suppresses killer lymphocyte-mediated pyroptosis in gastric cancer cells

**DOI:** 10.1371/journal.ppat.1014495

**Published:** 2026-08-05

**Authors:** Mingqian Xu, Shijia Hao, Shenlong Huang, Jihui Liu, Jiarui Lin, Junjie Zhang, Qingsong Qin

**Affiliations:** 1 Laboratory of Human Virology and Oncology, Shantou University Medical College, Shantou, Guangdong Province, China; 2 Department of Gynecologic Oncology, Cancer Hospital of Shantou University Medical College, Shantou, Guangdong Province, China; 3 Department of Gastrointestinal Surgery, Cancer Hospital of Shantou University Medical College, Shantou, Guangdong, China; 4 State Key Laboratory of Oral & Maxillofacial Reconstruction and Regeneration, Key Laboratory of Oral Biomedicine Ministry of Education, Hubei Key Laboratory of Stomatology, School & Hospital of Stomatology, State Key Laboratory of Virology and biosafety, Medical Research Institute, Wuhan University, Wuhan, China; Brigham and Women's Hospital, UNITED STATES OF AMERICA

## Abstract

Epstein-Barr virus (EBV)-associated gastric cancer (EBVaGC) is a distinct subtype of gastric cancer (GC) with characteristic clinicopathological and molecular features. EBVaGC exhibits a more extensive lymphocyte infiltration than EBV-negative counterparts, and expresses a range of EBV encoded viral products. However, the interactions between EBV-positive GC cells and immune cells within the tumor microenvironment are not well understood. Herein we found that EBV downregulates GSDMB, an executive molecule of pyroptosis, in EBV-positive GC cells and enables these cells to escape GSDMB cleavage-induced pyroptosis by NK cells and cytotoxic T lymphocytes. The pyroptosis is mainly executed through the full length GSDMB isoform (GSDMB^iso3^) in GC cells. We further found EBV-encoded miR-BART12-3p targets the GSDMB 3’-UTR to downregulate its expression in GC cells. Inhibition of miR-BART12-3p enhances antitumor efficacy of NK cells against EBV-positive GC cells by triggering GSDMB cleavage-induced pyroptosis, as demonstrated both *in vitro* and *in vivo*. In conclusion, this study elucidates the molecular mechanism by which EBV downregulates GSDMB to evade immune cell killing, and provides a rationale for combining killer lymphocyte-based therapies with a miR-BART12-3p inhibitor for the treatment of EBVaGC.

## Author summary

Epstein–Barr virus (EBV) infection is a known driver of a subset of gastric cancers (GCs). As a defense, immune cells can eliminate malignant cells through pyroptosis—a potent form of inflammatory programmed cell death. However, it remains unclear whether EBV-positive GC cells evade this pyroptotic attack by killer lymphocytes, and the mechanism behind such potential evasion has been poorly defined. In this study, we demonstrate that the EBV-encoded microRNA miR-BART12-3p plays a pivotal role in this immune escape process. We show that miR-BART12-3p directly targets the 3′-UTR of GSDMB mRNA to suppress its expression; GSDMB is a key executor of pyroptosis. By downregulating GSDMB, EBV-positive GC cells become resistant to lymphocyte-induced pyroptotic killing. Importantly, inhibiting miR-BART12-3p restores the sensitivity of EBV-positive GC cells to pyroptotic clearance. Our findings uncover a novel viral strategy for immune evasion and propose a potential therapeutic approach to enhance immunotherapy against EBV-associated gastric cancer.

## Introduction

EBV associated gastric cancer (EBVaGC) is classified as a distinct subtype of GC based on its clinicopathological and molecular features, accounts for approximately 9% of GC cases worldwide, making EBVaGC the largest group of EBV-associated malignancies [1,2]. The presence of EBV in GC cells and its distinct latent infection pattern underscore its crucial role in carcinogenesis and immune evasion [3]. However, the underlying mechanism has yet to be further studied. Compared to EBV-non-associated GCs (EBVnGCs), EBVaGCs upregulate immune regulatory genes that foster an immunosuppressive tumor microenvironment (TME), yet paradoxically display an “immune-hot” phenotype [4,5]. Notably, EBV positively correlates with enhanced infiltration of NK cells, CD8^+^ T cells, regulatory T cells, and macrophages [6]. To establish lifelong infections in the host, EBV has to overcome immune surveillance mechanisms. How EBV modulates the function of cytotoxic lymphocytes remains largely unknown in EBVaGC.

During latent infection, EBV employs restricted antigen expression to favor its survival and evade the host immune responses. EBV is known to encode microRNAs [7], and it utilizes BART miRNAs to maintain latency and facilitate viral escape from host immune surveillance by downregulating viral and host genes. For example, EBV miRNAs downregulate LMP1, reducing the expression of costimulatory molecules and MHC complexes on the cell surface, thereby impairing antigen presentation [8]. Specifically, miR-BART17 directly targets TAP2, an antigen peptide transporter, and suppresses EBNA1 expression, further disrupting viral antigen presentation [9]. MiR-BART1/2/22 suppress IL-12 to modulate inflammatory cytokine-driven immune responses [10]. While cytotoxic lymphocytes eliminate malignant cells via Fas/FasL interactions, perforin/granzyme pathways [11,12], and recently identified pyroptosis mechanisms [13], their ability to recognize HLA-deficient cancer cells underscores their importance in controlling gastric carcinogenesis [14]. It remains unclear whether EBV suppresses killer lymphocyte-mediated pyroptosis in GC.

Pyroptosis is mediated through the pores formation on cell membrane by the N-terminal domain of the gasdermin (GSDM) family proteins [15]. All GSDM members (except GSDMF, which lacks a C-terminal domain) consist of a N-terminal pore-forming domain, a C-terminal autoinhibitory domain, and an interdomain linker with variable length. Proteolytic cleavage within the linker region by caspases or other proteases releases the active N-terminal fragment, which binds membrane phospholipids and oligomerizes to form pores in the plasma membrane. This triggers rapid membrane rupture with concomitant release of cellular contents and proinflammatory mediators [16].

Members of GSDM family (D, E, B) are known to be activated by human caspases and granzymes through proteolytic cleavage. GSDMD, the first identified executor of pyroptosis, is cleaved by human caspases-1/4/5 or mouse caspases-1/11 to generate its pore-forming N-terminal fragment [16,17]. This fragment also exhibits direct bactericidal activity by disrupting bacterial membranes [18,19], highlighting its role in antimicrobial defense. GSDME, frequently silenced in cancers, can be re-expressed following treatment with DNA methyltransferase inhibitors such as decitabine [20]. Chemotherapeutic agents induce caspase-3-mediated GSDME cleavage, converting apoptosis to pyroptosis in tumor cells [21]. Cleavage of GSDME by granzyme B released from cytotoxic lymphocytes also leads to pyroptosis, which further enhances antitumor immunity by promoting macrophage phagocytosis and recruitment of NK/CD8^+^ T cells [22]. GSDMB (located at the chromosome 17q21) encodes six isoforms with differing linker regions and subcellular localization patterns [23]. Polymorphisms in GSDMB are associated with asthma and inflammatory bowel diseases [24]. Different proteases (granzyme A, elastase, and caspases) cleave GSDMB at different sites, which generate divergent effects on pyroptosis induction [25]. Among the cleavable isoforms, only isoforms 3 and 4 generate a pore-forming N-terminal domain upon cleavage by granzyme A released from cytotoxic lymphocytes [26,27]. The clinical implications of GSDMB are complex and context-dependent. For example, GSDMB isoform 2, which is dominantly expressed in HER2+ breast cancers, is associated with poorer outcomes [28,29]. While the high expression of GSDMB in other malignancies, such as bladder, skin, and kidney cancers, correlates with a more favorable prognosis based on TCGA data [13], dominant isoforms in these malignancies are not well defined.

In this study, we found that EBV-positive GC evades killer lymphocyte-mediated pyroptosis, which is regulated by the major pyroptosis-competent isoform of GSDMB (GSDMB^iso3^) in GC cells. We identified EBV-encoded miR-BART12-3p as a direct regulator that suppresses the expression levels of GSDMB in GC cells. Accordingly, inhibition of miR-BART12-3p rescues GSDMB expression and consequently sensitize EBVaGC to NK cell-mediated pyroptotic killing. These findings elucidate how EBV subverts killer lymphocyte-mediated antitumor immune surveillance, and reveal a novel therapeutic strategy for EBVaGC.

## Results

### EBV-positive GC cells escaping from NK cell-mediated pyroptosis is correlated with the downregulation of GSDMB

**Fig 1:**
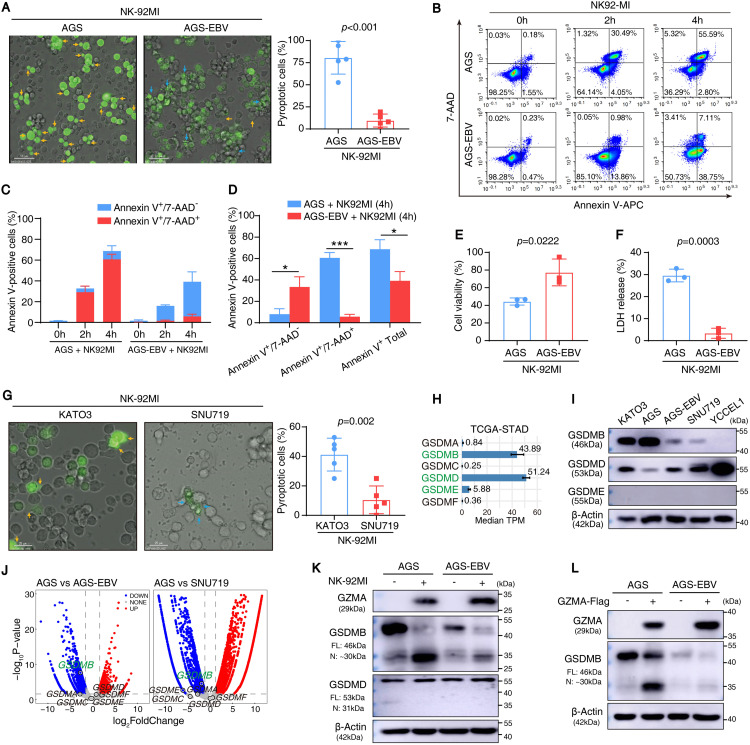
EBV-positive GC cells escaping from NK cell-mediated pyroptosis is correlated with the downregulation of GSDMB. **(A)** Time-lapse live-cell imaging of EBV-negative AGS and EBV-positive AGS-EBV gastric cancer cells pre-stained with calcein-labeled (green) during co-culture with NK-92MI cells (scale bars: 50 µm). Yellow arrows indicate characteristic pyroptotic features (cell swelling and membrane ballooning), while blue arrows mark apoptotic blebbing or non-pyroptotic cell death. Quantification of pyroptotic cells after co-culture is shown in the bar graph (n = 5). **(B)** Representative flow cytometry dot plots showing Annexin V/7-AAD staining of AGS and AGS-EBV cells at 0, 2, and 4 hours after co-culture with NK-92MI cells. The x-axis represents Annexin V-APC fluorescence, and the y-axis represents 7-AAD fluorescence. **(C)** Time-course quantification of Annexin V-positive populations (Annexin V^+^/7-AAD^-^ and Annexin V^+^/7-AAD^+^) in AGS and AGS-EBV cells after co-culture with NK-92MI cells for 0, 2, and 4 hours (n = 3). **(D)** Flow cytometry-based quantification of Annexin V-positive cell death in AGS and AGS-EBV cells at the 4-hour time point, stratified by early apoptotic (Annexin V^+^/7-AAD^-^), pyroptosis (Annexin V^+^/7-AAD^+^), and total Annexin V-positive populations (n = 3). **(E)** Cell viability of AGS and AGS-EBV cells after 4-hour co-culture with NK-92MI cells, measured by CCK-8 assay (n = 3). **(F)** Lactate dehydrogenase (LDH) release assay assessing lytic cell death in AGS versus AGS-EBV cells following 4-hour NK-92MI co-culture (n = 3). **(G)** Time-lapse live-cell imaging of EBV-negative KATO3 and naturally EBV-positive SNU719 GC cells pre-stained with calcein-labeled (green) during NK-92MI co-culture (scale bars: 25 µm). Quantification of pyroptotic cells is shown in the bar graph (n = 5). **(H)** Relative expression levels of gasdermin family members (GSDMA, GSDMB, GSDMC, GSDMD, GSDME) in gastric cancer tissues from the TCGA-STAD database, presented as median transcript per million (TPM) values. **(I)** Western blot analysis of GSDMB, GSDMD, and GSDME protein expression in EBV-negative GC cell lines (KATO3, AGS) and EBV-positive GC cell lines (AGS-EBV, SNU719, YCCEL1). β-Actin served as loading control. **(J)** Volcano plots showing differentially expressed genes between EBV-positive (AGS-EBV, SNU719) and EBV-negative (AGS) GC cells from RNA-sequencing data. Genes with a fold change ≥1.5 are shown; downregulated genes in EBV-positive cells are colored blue, upregulated genes red. Key pyroptosis-related gasdermin family genes are annotated. **(K)** Western blot analysis of GZMA, GSDMB, and GSDMD cleavage in AGS and AGS-EBV cells after 6-hour co-culture with NK-92MI cells. Full-length (FL) and cleaved (N-terminal, N) products are indicated. β-Actin served as loading control. **(L)** Western blot detection of GSDMB cleavage in AGS and AGS-EBV cells 48 hours after transfection with GZMA-Flag plasmid. β-Actin served as loading control. Data represent mean ± SD from at least three independent experiments. Statistical significance was determined by Student’s t-test (*P < 0.05, **P < 0.01 and ***P < 0.001).

EBV-associated gastric cancer (EBVaGC) is characterized with the extensive infiltration of lymphocytes in its microenvironment compared with EBV-negative gastric cancer (EBVnGC) [30]. To determine if EBV modulates the function of cytotoxic lymphocytes, we first assessed the NK cell cytotoxicity against EBV-negative (AGS, a human gastric adenocarcinoma cell line) and EBV-positive (AGS-EBV) GC cells via live-cell imaging. Following co-culture with NK cells, AGS cells exhibited hallmark features of pyroptosis (e.g., cellular swelling and membrane disruption) within 1 hour (Fig 1A, left panel, indicated by yellow arrow; S1 Movie). By contrast, following 7 ~ 8 hours of co-culture, AGS-EBV cells predominantly exhibited features of apoptosis, such as cell shrinkage and apoptotic body formation (right panel in Fig 1A, blue arrows; S2 Movie). We found typical pyroptotic features, such as cell swelling and pronounced membrane ballooning, were more frequently observed in AGS cells than in AGS-EBV cells.

To further quantify NK cell-induced cell death and characterize the mode of cell death in these two cell lines, we performed flow cytometry analysis using Annexin V/7-AAD staining at 0, 2, and 4 hours of co-culture. Consistent with the live-cell imaging results, the dot plots revealed strikingly distinct patterns of cell death progression between AGS and AGS-EBV cells (Fig 1B). In AGS cells, co-culture with NK cells led to a rapid expansion of the Annexin V^+^/7-AAD^+^ population (representing pyroptotic cells). The Annexin V^+^/7-AAD^-^ population (representing early apoptotic cells), by comparison, remained very low throughout the time course. In stark contrast, AGS-EBV cells exhibited a markedly different response. While the total Annexin V-positive population increased over time, the dominant population was Annexin V^+^/7-AAD^-^ early apoptotic cells. Quantitative analysis of these time-course data confirmed that the total proportion of Annexin V-positive cells increased with co-culture duration in both groups, but the magnitude and pattern differed dramatically (Fig 1C). At the 4-hour time point, stratification of the death populations revealed that AGS cells harbored a significantly higher proportion of Annexin V^+^/7-AAD^+^ pyroptotic cells, whereas AGS-EBV cells showed a significantly higher proportion of Annexin V^+^/7-AAD^-^ early apoptotic cells (Fig 1D). In addition, we observed that caspase-3 and PARP cleavage were more obvious in AGS-EBV cells and confirmed that these cells predominantly underwent apoptosis, whereas AGS cells did not (S1 Fig). Notably, the total percentage of Annexin V-positive cells (combining both populations) was significantly lower in AGS-EBV cells than in AGS cells at 4 h, indicating reduced overall susceptibility to NK cell-mediated killing (Fig 1D). Consistent with these results, after a 4-hour co-culture of GC cells with NK cells, AGS-EBV showed higher cell viability and lower LDH release than AGS cells (Fig 1E, 1F). This finding is consistent with the morphological evidence (Fig 1A), and further confirms that AGS-EBV cells are resistant to NK cell-mediated pyroptotic killing. Similar results were recapitulated when the EBV-negative GC cell line KATO3 or the naturally EBV‑positive GC cell line SNU719 was co‑cultured with NK cells (Fig 1G, S3 and S4 Movies). Collectively, these data demonstrate that EBV-positive GC cells evade NK cell mediated pyroptotic killing.

Pyroptosis is executed through activation of gasdermin (GSDM) family proteins [15]. To investigate the mechanism of NK cell-mediated pyroptosis in AGS cells, we first analyzed TCGA database, which revealed that GSDMB, -D, and -E are expressed in GC tissues (Fig 1H). GSDMB was previously reported to mediate pyroptosis in esophageal and colorectal cancer cells through cleavage by granzyme A (GZMA) released from NK cells [13], and recent studies have further elucidated the mechanism and significance of exosite-mediated targeting of GSDMB by dimeric granzyme A in lymphocyte pyroptotic killing [31]. In addition, granzyme B (GZMB) released by NK cells can triggers pyroptosis in both tumor cells (via caspase-3/GSDME pathway) and in virus-infected cells (via caspase-8/GSDMD pathway) [22,32]. We further examined the expression of GSDMB, GSDMD, and GSDME by Western Blotting. The results indicated that the levels of GSDMB protein in EBV-positive GC cells (AGS-EBV, SNU719, and YCCEL1) were lower than those in EBV-negative GC cells (AGS and KATO3) (Fig 1I). RNA-seq data also showed that GSDMB transcript levels were lower in AGS-EBV and SNU719 than in AGS (Fig 1J). Furthermore, we found that upon co-culture with NK cells, GSDMB in AGS cells was cleaved by GZMA, generating a 30 kDa N-terminal fragment capable of forming pores on the cell membrane. In contrast, the level of this cleaved GSDMB fragment was substantially lower in AGS-EBV cells (Fig 1K). Consistent with this, direct overexpression of GZMA in GC cells recapitulate the cleavage (Fig 1L). Together, these results indicate EBV-positive GC cells resist NK cell-mediated pyroptosis, likely through downregulation of GSDMB.

To validate our findings in a more physiologically relevant setting, we performed experiments using primary human NK cells isolated from peripheral blood mononuclear cells (PBMCs) of three independent healthy donors. Flow cytometric analysis confirmed high purity of the isolated NK cells (defined as CD3^-^CD56^+^ lymphocytes) (S2A Fig). Consistent with the results obtained with the NK-92MI cell line, primary NK cells induced significantly greater cytotoxicity in AGS cells compared to AGS-EBV cells after 4-hour co-culture at an effector-to-target ratio of 20:1 (S2B Fig). This enhanced susceptibility was observed across two of the three donors, as reflected by markedly reduced cell viability in AGS cells. Correspondingly, LDH release assays revealed significantly higher levels of lytic cell death in AGS cells compared to AGS-EBV cells after 4-hour co-cultured with primary NK cells from the same donors (S2C Fig). To determine whether the mechanism underlying this difference involves GSDMB cleavage, we performed western blot analysis on cell lysates collected after 6-hour co-culture with primary NK cells. We detected robust cleavage of full-length GSDMB into its ~30 kDa N-terminal fragment in AGS cells co-cultured with primary NK cells from all three donors (S2D Fig). In contrast, only minimal or no cleaved GSDMB was detectable in AGS-EBV cells under the same conditions, despite comparable levels of GZMA delivery. These data confirm that primary human NK cells preferentially trigger GSDMB-dependent pyroptotic killing in EBV-negative gastric cancer cells, while EBV-positive cells evade this mechanism, consistent with our observations using the NK-92MI cell line.

### EBV-positive GC cells resist GSDMB cleavage-mediated pyroptotic killing by CD8^+^ T cells

**Fig 2:**
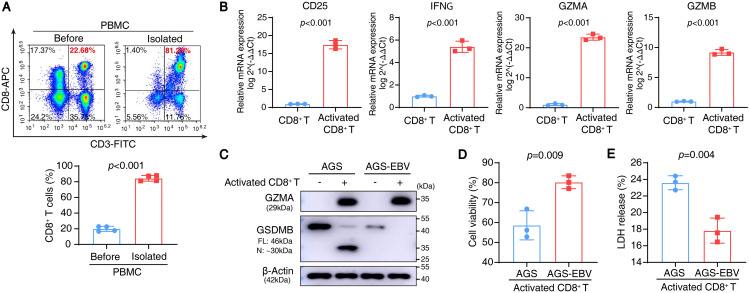
EBV-positive GC cells also resist GSDMB cleavage-mediated pyroptotic killing by CD8^+^ T cells. **(A)** The percentage of CD8⁺ T cells was evaluated by flow cytometry following isolation from human PBMCs. **(B)** After co-stimulation with anti-human CD3 and anti-human CD28 antibodies, the activation markers (CD25 and IFNγ) and cytotoxicity markers (GZMA and GZMB) of CD8⁺ T cells were detected by RT-qPCR. **(C)** Western blot was used to detect the cleavage products of GSDMB in GC cells after 6-hour co-culture with CD8⁺ T cells. β-Actin served as loading control. **(D)** Cell viability between AGS and AGS-EBV cells compared by CCK-8 assay after 4-hour co-culture with CD8⁺ T cells (n = 3). **(E)** LDH release assay demonstrating lytic cell death in AGS versus AGS-EBV cells post 4-hour co-incubation with CD8⁺ T cells (n = 3). Data represent mean ± SD from three independent experiments. Statistical significance determined by two-tailed Student's t-test (*P* < 0.05; *P < 0.01; **P < 0.001).

Since cytotoxic T lymphocytes (CTLs) can also release perforin and granzyme A, we further investigated whether EBV-positive GC also resist the cytotoxic T cell mediated pyroptotic killing. As CD8^+^ T cells isolated from healthy donors are unable to kill GC cells through T cell receptor recognition and targeting, isolated CD8^+^ T cells were activated with anti-human CD3 and CD28 antibodies. CD8⁺ T cells were isolated from human PBMCs and assessed by flow cytometry (Fig 2A). Subsequently, isolated CD8⁺ T cells were activated by co-stimulation with anti-human CD3 and anti-human CD28 antibodies, and the transcript levels of activation markers (CD25, IFNG, GZMA, GZMB) were quantified via RT-qPCR (Fig 2B). Then the activated cytotoxic CD8⁺ T cells were co-cultured with AGS or AGS-EBV GC cells at an E/T ratio of 10. Western blotting of 6-hour co-cultured cell lysates revealed GZMA-mediated GSDMB cleavage in AGS cells. In contrast, the cleaved form of GSDMB was barely detected in AGS-EBV cells (Fig 2C). Furthermore, in comparison to AGS, AGS-EBV cells resisted CD8⁺ T cell-mediated pyroptotic killing, as evidenced by CCK-8 assays (Fig 2D) and LDH release assay (Fig 2E). Based on these findings, we hypothesize that EBV infection promotes immune evasion by inducing GSDMB deficiency to confer pyroptosis resistance, enabling tumor cells to evade antitumor immunity mediated by cytotoxic lymphocytes.

### Knockdown of GSDMB inhibits cytotoxic lymphocytes mediated pyroptosis in EBV-negative GC cells

**Fig 3:**
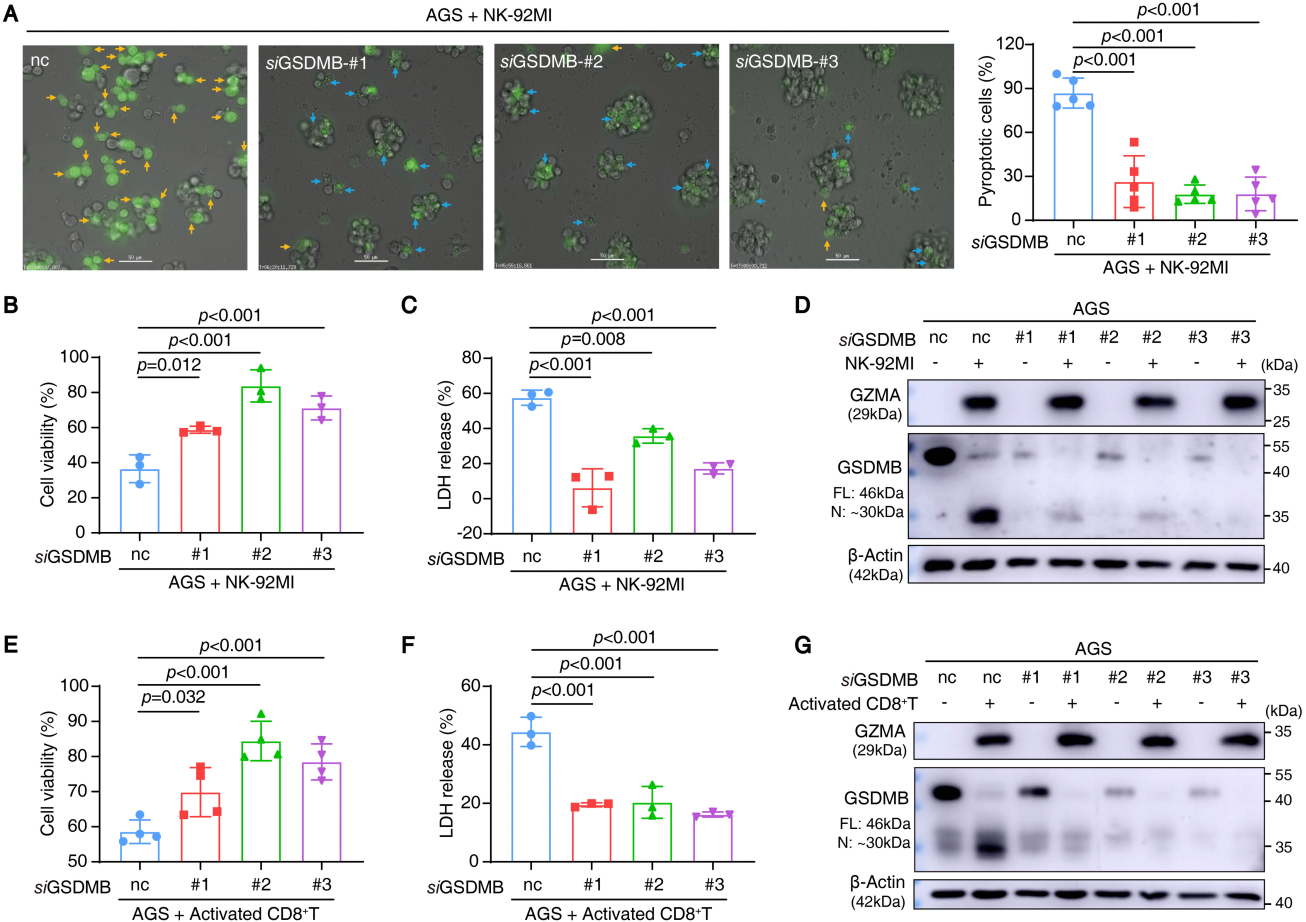
Knockdown of GSDMB inhibits cytotoxic lymphocytes mediated pyroptosis in EBV-negative GC cells. **(A)** Time-lapse microscopy monitored the type of cell death of AGS cells transfected with GSDMB siRNA or its corresponding control siRNA and pre-stained with calcein-labeled (green) during NK cell interaction (scale bars: 50 μm; yellow arrows indicate characteristic bubble-like plasma membrane protrusions and blue arrows indicate apoptotic blebbing or non-pyroptotic cell death), and pyroptotic cells (%) during co-culture with NK cells was quantified by morphology-based analysis (n = 5). **(B)** Cell viability assessment by CCK-8 assay following 4-hour of co-culture with NK cells (n = 3). **(C)** LDH release assay demonstrating lytic cell death following 4-hour NK cells co-culture (n = 3). **(D)** Western blot analysis of GSDMB expression and N-terminal cleavage products in AGS cells transfected with GSDMB-specific siRNAs versus scramble control. **(E)** CCK-8 viability assay and **(F)** LDH release assay post 4-hour CD8⁺ T cells interaction (n = 3). **(G)** Western blot analysis of GSDMB expression and N-terminal cleavage products in AGS cells transfected with GSDMB-specific siRNAs versus scramble control following co-culture with CD8⁺ T cells. β-Actin served as loading control. Data represent mean ± SD from three independent experiments. Statistical significance determined by one-way ANOVA (analysis of variance) analysis (*P < 0.05, **P < 0.01, ***P < 0.001).

To further examine if GSDMB mediates pyroptosis in AGS cells, we knock it down using specific siRNAs. Using live-cell imaging, we observed a substantially decrease in NK cell-induced pyroptotic cell death (Fig 3A). Concomitantly, the cells resisted to NK cell-mediated killing (Fig 3B) and exhibited attenuated lytic cell death, as evidenced by reduction in LDH release (Fig 3C). Consistently, the cleaved form of GSDMB was barely detected following co-culture with NK cells (Fig 3D). Furthermore, knockdown of GSDMB rendered AGS cells resistant to activated CD8⁺ T cell-mediated cytotoxicity (Fig 3E-3F). This resistance correlated to nearly undetectable levels of cleaved GSDMB following co-culture (Fig 3G). Together, these results suggest that GSDMB is the pyroptotic executor molecule in GC cells.

### Overexpressing GSDMB^iso3^ promotes NK cell-mediated pyroptosis in EBV-positive GC cells

**Fig 4:**
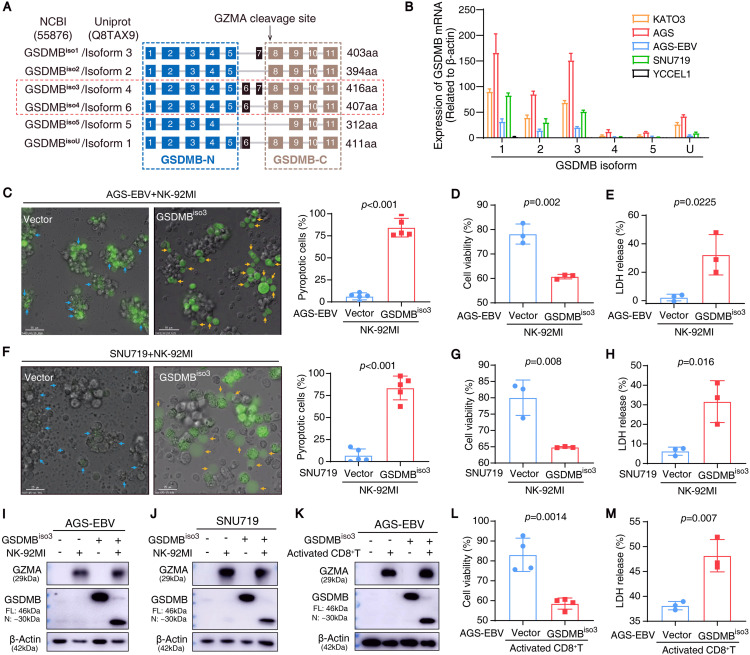
Overexpressing GSDMB^iso3^ promotes NK cell-mediated pyroptosis in EBV-positive GC cells. **(A)** Schematic of six GSDMB splicing isoforms. **(B)** RT-qPCR quantification of GSDMB isoforms in EBV-negative (KATO3, AGS) and EBV-positive (AGS-EBV, SNU719 and YCCEL1) GC cells. **(C)** Time-lapse imaging of AGS-EBV cells transfected with GSDMB^iso3^ or empty vector and pre-stained with calcein-labeled (green) during NK co-culture (scale bars: 50 μm; yellow arrows indicate pyroptotic rupture and blue arrows indicate apoptotic blebbing or non-pyroptotic cell death). The quantification of pyroptotic cells following incubation with NK cells is presented in a column chart (n = 5). **(D)** CCK-8 viability assay and **(E)** LDH release assay post 4-hour NK cells interaction (n = 3). **(F)** Time-lapse imaging of SNU719 cells transfected with GSDMB^iso3^ or empty vector and pre-stained with calcein-labeled (green) during NK co-culture (scale bars: 50 μm). The quantification of pyroptotic cells following incubation with NK cells is presented in a column chart (n = 5). **(G)** CCK-8 viability assay and **(H)** LDH release assay post 4-hour NK cells interaction (n = 3). **(I-J)** Western blot analysis of GSDMB cleavage in EBV-positive GC cells transfected with GSDMB^iso3^ or empty vector after 6-hour NK cells co-culture. β-Actin served as loading control. **(K)** Western blot analysis of GSDMB cleavage in AGS-EBV cells transfected with GSDMB^iso3^ or empty vector after CD8⁺ T cells co-culture. β-Actin served as loading control. **(L)** Cell viability assessment by CCK-8 assay following co-culture with CD8⁺ T cells (n = 3). **(M)** LDH release assay demonstrating lytic cell death post CD8⁺ T cells co-culture (n = 3). Data represent mean ± SD from three independent experiments. Statistical significance determined by two-tailed Student's t-test (*P* < 0.05; *P < 0.01; **P < 0.001).

Six GSDMB isoforms were annotated in public databases (NCBI and UniProt), as illustrated in Fig 4A. Alternative splicing of exons 6 and 7 produces GSDMB isoforms 1–4, all containing GZMA cleavage sites, among which only isoforms 3 and 4 retain exon 6 and thus maintain the functional pore-forming domains capable of inducing pyroptosis [26,27]. Using isoform-specific qPCR primers (Table 1), we quantified GSDMB isoforms in EBV-positive and EBV-negative GC cells. We found while all isoforms were detectable in both cell types, GSDMB^iso3^ was expressed at markedly higher levels than GSDMB^iso4^ (Fig 4B), we therefore focus subsequent studies on GSDMB^iso3^.

**Table 1:** Sequence of GSDMB specific qPCR primers.

Name	Sense (5’-3’)
isoform 1 (F)	AAGGAGACGATGAAGAAGGATGGT
isoform 1 (R)	TTCTCTTCTCCTCTGTCAGGTCCT
isoform 2 (F)	GGAGACGATGAGAAAGTCTTTGGGT
isoform 2 (R)	TTCTCTTCTCCTCTGTCAGGTCCT
isoform 3 (F)	ATCCTTTCCAGAAGAGAAGGATGGT
isoform 3 (R)	TTCTCTTCTCCTCTGTCAGGTCCT
isoform 4 (F)	AATCCTTTCCAGAAGGAAAGTC
isoform 4 (R)	TTCTCTTCTCCTCTGTCAGGTC
isoform 5 (F)	GAAACCCTGAAAAGCGACCG
isoform 5 (R)	CAGGACCTCAGATACCTTGTGT
isoform U (F)	CGATGAGTGCTGGTTTGGATATTCA
isoform U (R)	TTCTCTTCTCCTCTGTCAGGTCCT

To interrogate the functional role of GSDMB^iso3^ in EBV-positive GC cells, we generated isoform 3-specific expression plasmids. Overexpression of GSDMB^iso3^ enhanced the occurrence of NK cell-mediated pyroptosis in EBV-positive GC cells, as assessed by live-cell imaging (Fig 4C and 4F, S5-S8 Movies). Consistently, elevated GSDMB^iso3^ expression in EBV-positive GC cells led to more potent NK cell-mediated cytotoxicity (Fig 4D and 4G) and higher LDH release (Fig 4E and 4H) following 4-hour co-culture. Consistent with this pro-pyroptotic function, western blot analysis showed increased generation of 30-kDa N-terminal cleavage fragments in EBV-positive GC cells after a 6-hour co-culture with NK cells (Fig 4I-4J). Similarly, overexpression of GSDMB^iso3^ in AGS-EBV cells yielded GZMA-cleaved GSDMB fragments upon co-culture with activated CD8⁺ T cells (Fig 4K) and enhanced their susceptibility to CD8⁺ T cell-induced pyroptosis, which was evidenced by enhanced cytotoxicity (Fig 4L) and increased LDH release (Fig 4M). Collectively, these results further establish GSDMB^iso3^ as a major pyroptosis executor in GC cells for cytotoxic lymphocyte-mediated killing.

### EBV-encoded miR-BART12-3p directly targets the 3’-UTR of GSDMB transcripts to down-regulate the expression of GSDMB

**Fig 5:**
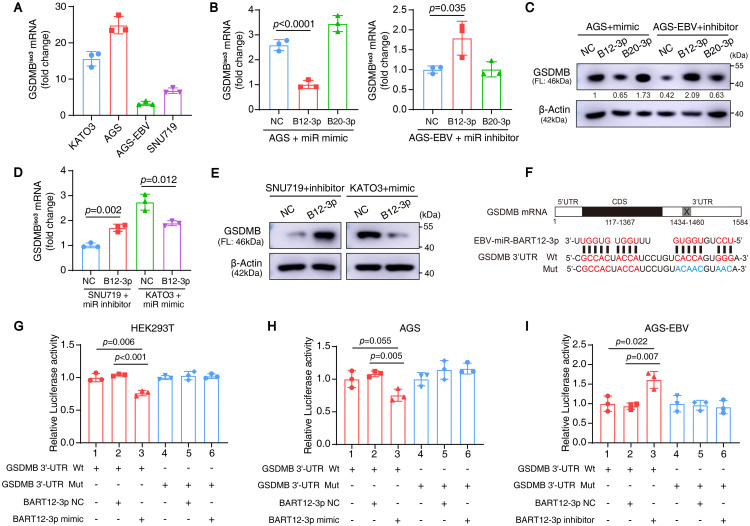
EBV-encoded miR-BART12-3p directly targets the 3’-UTR of GSDMB transcripts to down-regulate the expression of GSDMB. **(A)** *GSDMB* isoform 3 mRNA levels in EBV-negative (KATO3, AGS) versus EBV-positive (AGS-EBV, SNU719) GC cells by RT-qPCR. **(B)** *GSDMB* isoform 3 transcriptional suppression by miR-BART12-3p / miR-BART20-3p mimics (left) and restoration by their inhibitors (right) using RT-qPCR. **(C)** Western blot analysis demonstrating miR-BART12-3p-mediated GSDMB protein downregulation (β-Actin loading control). **(D-E)** Following miR-BART12-3p inhibition in SNU719 (EBV^+^ GC) cells and miR-BART12-3p overexpression in KATO3 (EBV^-^ GC) cells, *GSDMB* isoform 3 mRNA **(D)** and **(E)** protein levels were respectively detected through RT-qPCR and western blot analysis. **(F)** Predicted miR-BART12-3p binding sites in wild-type (Wt) *GSDMB* 3’-UTR and seed region-mutated (Mut) constructs. **(G-I)** Dual luciferase reporter assays: **(G)** HEK293T cells or **(H)** AGS cells were co-transfected with a Wt or Mut GSDMB 3’-UTR luciferase reporter vector and BART12-3p mimic or its control mimic, while **(I)** AGS-EBV cells were co-transfected with a Wt or Mut GSDMB 3’-UTR luciferase reporter vector and BART12-3p inhibitor or its control inhibitor, and then luciferase activity was detected 48h after transfection. Data are presented as mean ± standard deviation (*P < 0.05, **P < 0.01, ***P < 0.001).

Next, we sought to explore how EBV infection down-regulated the expression of GSDMB in GC cells. EBV expresses high levels of EBV-encoded BART miRNAs that play multiple functions towards tumorigenesis, and avoid triggering immune response at the same time [33]. RT-qPCR analysis showed that the transcription levels of GSDMB^iso3^ were lower in EBV-positive GC cells (Fig 5A), which was supported by western blot analysis and RNA sequencing data (Fig 1I-1J). Thus, we hypothesized that EBV BART miRNAs mediate post-transcriptional downregulation of GSDMB. Using RNAhybrid (https://bibiserv.cebitec.uni-bielefeld.de/rnahybrid) for prediction, we identified miR-BART12-3p and miR-BART20-3p as top candidate miRNAs targeting the GSDMB transcript (Table 2). Functional validation showed that overexpression of miR-BART12-3p in AGS cells decreased GSDMB^iso3^ mRNA levels, whereas inhibition of miR-BART12-3p in AGS-EBV cells elevated them. In contrast, manipulation of miR-BART20-3p (overexpression or inhibition) did not affect the mRNA levels of GSDMB^iso3^ (Fig 5B). The regulatory impact of miR-BART12-3p was further confirmed at the protein levels (Fig 5C) and reproducible in SNU719 and KATO3 cells (Fig 5D-5E). Mechanistically, dual luciferase reporter assays confirmed direct binding of miR-BART12-3p to the GSDMB 3’-UTR. Introduction of seed region mutations (Fig 5F) abolished miR-BART12-3p-mediated suppression of reporter gene expression in HEK293T cells as well as AGS cells (Fig 5G-5H). Accordingly, the repressive activity of miR-BART12-3p was negated, leaving luciferase expression unaltered in AGS-EBV cells upon its inhibition (Fig 5I). We also examine the expression of GSDMB in HEK293T cells co-expressing GSDMB and miR-BART12-3p, and the results demonstrate that miR-BART12-3p significantly downregulates GSDMB protein expression compared to the negative control (S3 Fig). This finding is consistent with the luciferase reporter assay in HEK293T cells (Fig 5G), further supporting the conclusion that miR-BART12-3p directly targets GSDMB. These results establish EBV-encoded miR-BART12-3p as a post-transcriptional suppressor of GSDMB expression, enabling EBV-positive GC cells to evade NK cell-induced pyroptosis.

**Table 2:** Binding efficiency of GSDMB and 44 EBV-encoded BART miRNAs predicted by the RNAhybrid program.

Rank	miRNA name	ID number	length	position	mfe (kcal/mol)
1	ebv-miR-BART12-3p	MIMAT0003423	22	67	-31.0
2	ebv-miR-BART20-3p	MIMAT0003720	22	196	-30.4
3	ebv-miR-BART7-3p	MIMAT0003416	22	164	-27.9
4	ebv-miR-BART21-3p	MIMAT0010131	22	83	-27.6
5	ebv-miR-BART6-3p	MIMAT0003415	22	28	-25.2
6	ebv-miR-BART10-3p	MIMAT0003420	23	108	-24.7
7	ebv-miR-BART3-3p	MIMAT0003411	22	163	-24.0
8	ebv-miR-BART2-3p	MIMAT0004744	24	131	-23.6
9	ebv-miR-BART13-3p	MIMAT0003424	23	161	-23.5
10	ebv-miR-BART1-5p	MIMAT0000999	24	57	-22.9
11	ebv-miR-BART18-3p	MIMAT0004835	22	162	-22.8
12	ebv-miR-BART4-5p	MIMAT0009205	22	61	-22.5
13	ebv-miR-BART1-3p	MIMAT0003390	22	195	-21.9
14	ebv-miR-BART11-5p	MIMAT0003421	24	58	-21.8
15	ebv-miR-BART11-3p	MIMAT0003422	21	161	-21.4
16	ebv-miR-BART15-5p	novel	24	181	-21.4
17	ebv-miR-BART10-5p	MIMAT0004817	22	175	-21.2
18	ebv-miR-BART16	MIMAT0003714	24	67	-21.2
19	ebv-miR-BART5-5p	MIMAT0003413	24	30	-20.4
20	ebv-miR-BART6-5p	MIMAT0003414	22	78	-20.3
21	ebv-miR-BART8-3p	MIMAT0003418	23	181	-20.3
22	ebv-miR-BART22	MIMAT0010132	23	175	-20.2
23	ebv-miR-BART9-3p	MIMAT0003419	23	70	-19.7
24	ebv-miR-BART13-5p	MIMAT0004818	22	50	-19.5
25	ebv-miR-BART17-5p	MIMAT0003715	22	48	-19.5
26	ebv-miR-BART15	MIMAT0003713	22	86	-19.2
27	ebv-miR-BART9-5p	MIMAT0004816	22	22	-18.8
28	ebv-miR-BART4-3p	MIMAT0009204	23	163	-18.7
29	ebv-miR-BART20-5p	MIMAT0003719	21	195	-18.7
30	ebv-miR-BART12-5p	novel	21	165	-18.5
31	ebv-miR-BART18-5p	MIMAT0003717	22	196	-18.3
32	ebv-miR-BART17-3p	MIMAT0003716	23	70	-18.0
33	ebv-miR-BART14-3p	MIMAT0003426	22	96	-17.8
34	ebv-miR-BART19-5p	MIMAT0004836	23	80	-17.8
35	ebv-miR-BART21-5p	MIMAT0010130	21	47	-17.8
36	ebv-miR-BART5-3p	MIMAT0009205	18	179	-17.3
37	ebv-miR-BART2-5p	MIMAT0001000	22	194	-17.2
38	ebv-miR-BART3-5p	MIMAT0003410	21	7	-17.2
39	ebv-miR-BART8-5p	MIMAT0003417	22	21	-17.2
40	ebv-miR-BART22-5p	novel	22	82	-17.2
41	ebv-miR-BART14-5p	MIMAT0003425	22	191	-16.7
42	ebv-miR-BART19-3p	MIMAT0003718	21	128	-16.4
43	ebv-miR-BART7-5p	MIMAT0004815	22	47	-15.2
44	ebv-miR-BART16-3p	novel	21	178	-14.9

### Inhibition of miR-BART12-3p sensitizes EBV-positive GC cells to NK cell-mediated pyroptosis

**Fig 6:**
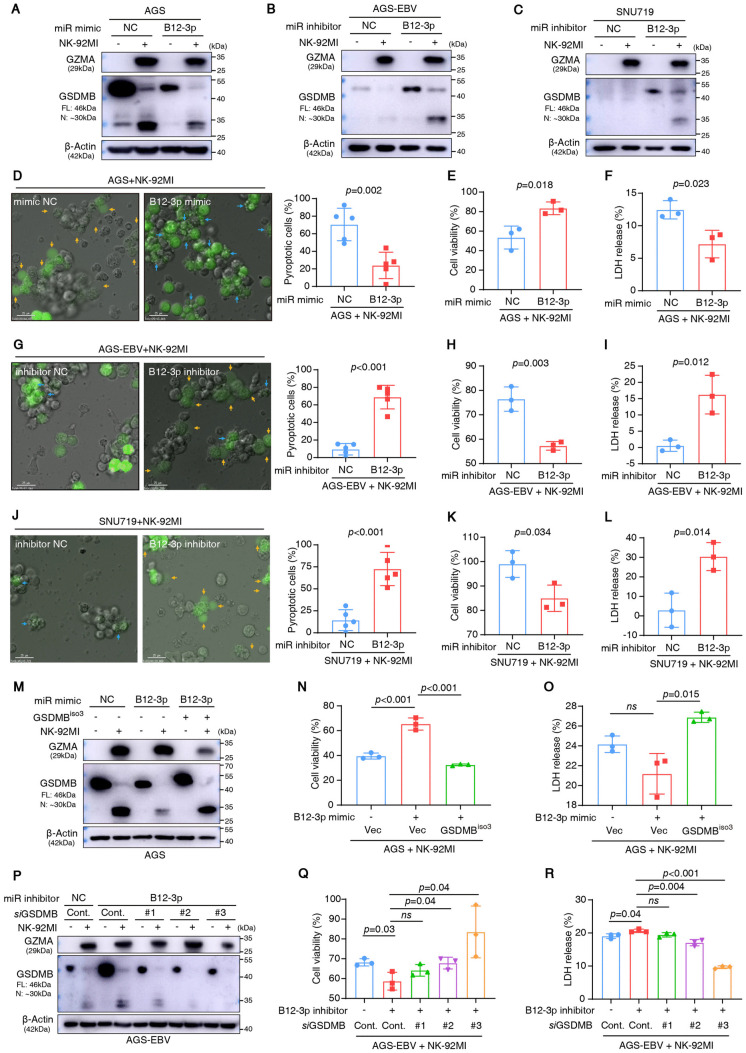
Inhibition of miR-BART12-3p sensitizes EBV-positive GC cells to NK cell-mediated pyroptosis. **(A-C)** Western blot analysis of GSDMB expression and cleavage in gastric cancer cells transfected with miR-BART12-3p mimic or inhibitor versus their corresponding control for 48h, followed by 6-hour NK cells co-culture. **(D)** Time-lapse imaging of pyroptotic morphology in AGS cells with or without miR-BART12-3p after co-culture with NK cells (scale bars: 25 μm). AGS cells were pre-stained with calcein-AM (green) and pyroptotic cells (%) during co-culture with NK cells was quantified by morphology-based analysis (n = 5). **(E)** CCK-8 cell viability assay and **(F)** LDH release assay in AGS cells after exposure to NK cells for 4h (n = 3). **(G)** Time-lapse imaging of pyroptotic morphology in AGS-EBV cells with or without miR-BART12-3p inhibition after co-culture with NK cells (scale bars: 25 μm). AGS-EBV cells were pre-stained with calcein-AM (green) and pyroptotic cells (%) during co-culture with NK cells was quantified by morphology-based analysis (n = 5). **(H)** CCK-8 cell viability assay and **(I)** LDH release assay in AGS-EBV cells after exposure to NK cells for 4h (n = 3). **(J-L)** Parallel analysis in SNU719 cells. **(M-O)** Rescue experiments in AGS cells: **(M)** GSDMB expression and cleavage in AGS cells after co-transfection of miR-BART12-3p mimic and GSDMB over-expression vector or their corresponding negative controls for 48h, followed by 6-hour NK cells co-culture (Western blots). **(N)** Cell viability (CCK-8) and **(O)** lytic cell death (LDH release) comparison post 4-hour NK cells interaction (n = 3). **(P-R)** Knockdown studies in AGS-EBV cells: **(P)** Western blot analysis of GSDMB expression and cleavage in AGS-EBV cells after co-transfection of miR-BART12-3p inhibitor and siRNAs or their corresponding negative controls for 48h, followed by 6-hour NK cells co-culture. **(Q)** Cell viability (CCK-8) and **(R)** lytic cell death (LDH release) comparison after exposure to NK cells for 4h (n = 3). Data represent mean ± SD. Statistical significance determined by Student’s t-test for two groups, and one-way ANOVA (analysis of variance) analysis for multiple groups (*P < 0.05, **P < 0.01, ***P < 0.001).

To functionally validate the role of miR-BART12-3p in EBV-associated immune evasion, we performed NK cell co-culture assays with GC cells transfected with BART12-3p mimic. We found that transfection of AGS cells with miR-BART12-3p mimic for 48 h, followed by 6 h co-culture with NK cells (Fig 6A) diminished pyroptotic morphological changes of AGS cells via live-cell imaging (Fig 6D, S9 and S10 Movies). Functionally, this corresponded to increased cell viability (Fig 6E) and reduced LDH release (Fig 6F). Collectively, these results confirm BART12-3p confers resistance to pyroptosis. Conversely, inhibition of miR-BART12-3p in EBV-positive GC cells (AGS-EBV and SNU719) increased GZMA-cleaved GSDMB upon co-culture with NK cells (Fig 6B-6C) and triggered rapid pyroptosis (Fig 6G and 6J; S11-S14 Movies), alongside boosted NK cell cytotoxicity (Fig 6H, 6I) and lytic activity (Fig 6K, 6L).

To establish a causal link between GSDMB suppression and miR-BART12-3p-mediated immune evasion in GC cells, we performed functional rescue assays in AGS cells. Reconstitution of GSDMB in AGS cells treated with miR-BART12-3p mimic (Fig 6M) effectively reversed the immune escape of miR-BART12-3p, as evidenced by enhanced NK cell cytotoxicity (Fig 6N) and increased lytic activity (Fig 6O). Conversely, the enhanced NK cell-mediated killing of AGS-EBV cells upon miR-BART12-3p inhibition was abrogated by GSDMB knockdown (Fig 6P-6R). Thus, we demonstrated that EBV exploits miR-BART12-3p to suppress GSDMB and subvert pyroptosis, thereby unveiling a new axis of viral immune evasion.

### miR-BART12-3p inhibitor potentiates NK cells mediated killing in EBV-positive GC cells *in vivo*

**Fig 7:**
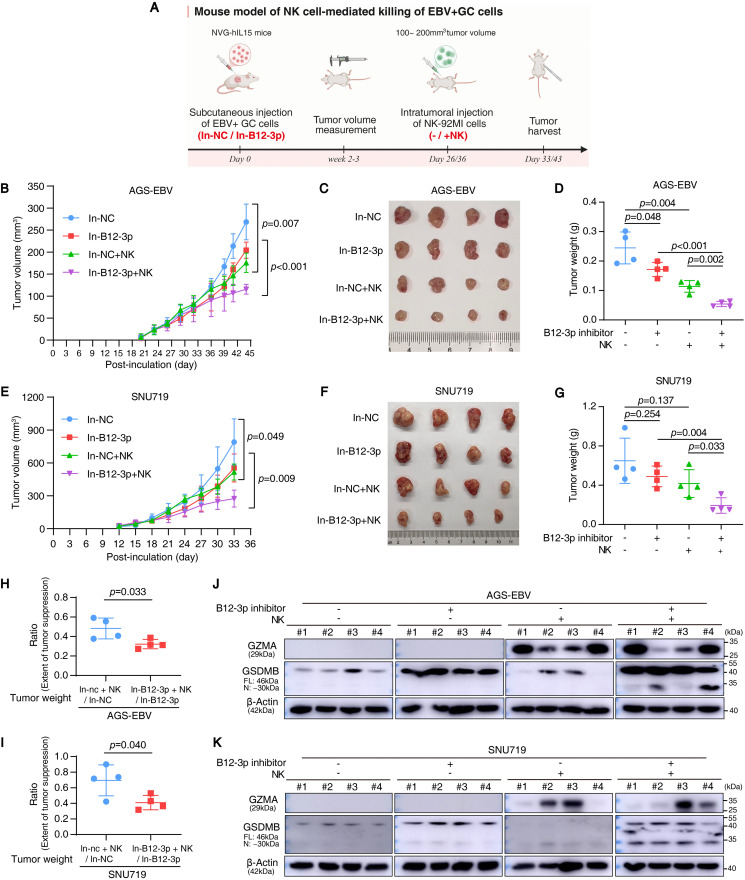
miR-BART12-3p inhibitor promotes NK cell-mediated GSDMB cleavage *in vivo* and controls EBV-positive gastric cancer growth. **(A)** Schematic of the *in vivo* protocol for evaluating NK cell-mediated tumor clearance following EBV-miR-BART12-3p inhibition in EBV-positive GC cells using NVG-hIL15 mice (Created in BioRender. Xu, M. (2026) https://BioRender.com/tajh660). After inhibition of miR-BART12-3p, two EBV-positive GC cells (AGS-EBV and SNU719) were subcutaneously inoculated into NVG-hIL15 mice (4 group, 4 mice/group). Tumor volume was periodically measured for each mouse and tumor growth curves were plotted. The xenograft was intratumorally injected with NK cells or normal saline when it reached 100–200 mm^3^. The experiments were terminated 7 days after the NK cell injection. **(B and E)** Tumor growth curves, **(C and F)** images of tumor xenografts and **(D and G)** tumor weight are shown. **(H-I)** Quantitative comparison of tumor weight ratio between NK-treated and untreated groups. The ratios of In-NC + NK / In-NC and In-B12-3p + NK / In-B12-3p were calculated and compared. **(J-K)** The protein levels of GZMA and GSDMB in tumor samples from mice in the groups studied above were detected by western blotting and normalized to the level of β-Actin. Data represent mean ± SD from three independent experiments. Statistical significance determined by two-tailed Student's t-test (*P* < 0.05; *P < 0.01; **P < 0.001).

To further determine whether miR-BART12-3p inhibitor can sensitize EBV-positive GC cells to NK cell-mediated killing *in vivo*, two EBV-positive GC cell lines (AGS-EBV and SNU719) were transfected with miR-BART12-3p inhibitor for 24h, and then subcutaneously transplanted into NVG-hIL15 mice. Upon the xenografts reached 100–200 mm^3^, NK cells were delivered via intratumoral injection. The experiments were designed to terminate 7 days after NK cell injection as illustrated in Fig 7A. The results showed that inhibition of miR-BART12-3p potentiated NK cell-mediated clearance of xenografts from both EBV-positive GC cell lines (Fig 7B-7G). To quantify NK cell-mediated tumor suppression, we calculated the ratios of tumor weight in the NK-treated groups relative to their respective untreated controls (i.e., In-NC + NK / In-NC and In-B12-3p + NK / In-B12-3p). The ratio of tumor weight was significantly higher in the In-NC group than that in the In-B12-3p group, indicating stronger NK cell-mediated growth inhibition in miR-BART12-3p-knockdown tumors (Fig 7H-7I). Western blotting analysis of xenografts further confirmed that miR-BART12-3p inhibitor led to accumulation of GZMA-induced GSDMB cleavage (Fig 7J-7K). Consistent with these GSDMB cleavage data, qRT-PCR analysis of harvested tumor tissues at the experimental endpoint confirmed that miR-BART12-3p expression remained at a significantly lower level in the In-B12-3p group than in the In-NC group (S4 Fig). This sustained low level of miR-BART12-3p expression likely underlies the enhanced GSDMB cleavage and improved NK cell-mediated tumor control observed *in vivo*. Collectively, these results suggest that targeting miR-BART12-3p offers a novel therapeutic strategy for EBVaGC by sensitizing tumor cells to cytotoxic lymphocyte-mediated pyroptosis.

## Discussion

**Fig 8:**
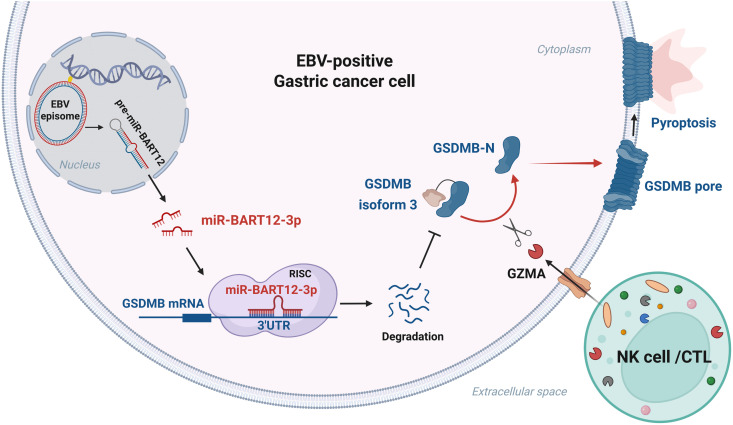
Proposed mechanism of EBV-mediated immune evasion through GSDMB suppression in gastric cancer. NK cells as well as cytotoxic T lymphocytes release GZMA to cleave GSDMB^iso3^ in EBV-negative GC cells, ultimately inducing tumor cells rapid pyroptosis. EBV subverts this antitumor immunity through viral miR-BART12-3p, which binds complementary sequences in the *GSDMB* 3’-UTR to suppress both mRNA stability and protein translation. This miRNA-mediated GSDMB downregulation in EBV-infected GC cells prevents GZMA-induced pore formation, thereby blocking pyroptotic clearance and enabling immune escape. (The figure was created in BioRender. Xu, M. (2026) https://BioRender.com/op9y3fj).

The host employs innate or adaptive immune systems to combat microbial or viral infections, while pathogens invariably devise strategies to evade immune surveillance. In this study, we reveal a clear dichotomy in gastric cancer cell fate upon killer lymphocyte attack: EBV-negative GC cells undergo rapid inflammatory pyroptosis, whereas EBV-positive GC cells experience slow non-inflammatory apoptosis. Mechanistically, we demonstrated EBV-encoded miR-BART12-3p directly targets the 3’-UTR of GSDMB transcript to downregulate the expression of GSDMB, thereby allowing GC cells to evade pyroptosis induced by granzyme A released from killer lymphocytes (NK cells and cytotoxic T lymphocytes) as illustrated in (Fig 8).

This study provides novel insights into the immune evasion mechanisms in EBVaGC. Cytotoxic lymphocytes kill tumor cells through two distinct programed cell death: apoptosis and pyroptosis. Apoptosis is a non-inflammatory programmed cell death marked by cell shrinkage, intact plasma membranes, and no release of cellular content. In contrast, pyroptosis features cell swelling, plasma membrane rupture, and the release of inflammatory cytokines [13]. Cytotoxic lymphocytes mediated pyroptosis not only kills tumor cells rapidly also stimulates robust anti-tumor immunity and converts immunologically inert tumors into immunologically active microenvironments [34]. As immune escape represents one of the established hallmarks of cancer progression [35], EBV-encoded miRNAs target host genes involved in immune evasion by NK cells and T lymphocytes. For example, miR-BART2-5p maintains tumor cell survival by downregulating the major histocompatibility complex (MHC) class I polypeptide-related sequence B (MICB) recognized by the natural killer group 2 member D receptor present on NK cells [36]. miR-BHRF1–3-5p and miR-BART17-5p target transporter associated with antigen processing 2 (TAP2) that transports antigenic peptides to MHC class I molecules, thus, viral antigen presentation is impaired in CD8^+^ T cells [9,37]. In this study, we found that miR-BART12-3p directly targets and downregulates GSDMB isoform 3, the major pyroptosis executor in GC cells, thereby conferring resistance to cytotoxic lymphocyte-mediated granzyme A-induced and GSDMB-cleavage dependent pyroptotic death, which provides new evidence that EBV-encoded miRNAs modulate pyroptosis mediated by NK cells and T lymphocytes. EBV-encoded miR-BART12-3p was abundantly expressed in EBVaGC tissues and cell lines [38–40], and reported to display dual functional roles in carcinogenesis: it inhibits proliferation and migration by targeting Snail, and suppresses microtubule polymerization protein 1 (TPPP1) to promote migration and invasion [41,42].

GSDMB isoforms differ in pore-forming activity [43], therefore the alternative splicing of GSDMB also modulates pyroptosis mediated by cytotoxic lymphocytes. Initial studies showed that NK cell-derived granzyme A cleaves GSDMB, leading to pore formation and pyroptotic death [13]. Further studies revealed a non-pyroptotic role for GSDMB in promoting epithelial repair in inflammatory bowel disease [44]. This controversy was largely resolved by cryo-EM structural analyses showing that exon 6-mediated variations in the interdomain linker region dictate the membrane binding and oligomerization capacity of the N-terminal domain, thereby explaining divergent pore-forming activities [45,46]. Consequently, only isoforms 3 and 4 exhibit pyroptotic activity due to their intact pore-forming domains. Prognostic associations differ by isoform: high expression of isoform 2 correlates with poor outcomes in breast and renal cancers, whereas upregulation of isoform 3 predicts favorable survival in bladder cancer [25,43]. As shown in Fig 4B, pyroptosis in GC cells depends on the presence of pyroptosis-active isoforms. In breast cancer, overexpression of isoforms 1, 2, or 3 enhances cell motility and invasion [29,47], with isoform 2 specifically promoting tumor growth, metastasis, and HER2/NEU-driven carcinogenesis [25,46,47]. GSDMB upregulation also facilitates bladder cancer progression by modulating glycolytic metabolism [48], though isoform specificity was unaddressed. Tumor cells often co-express pyroptosis-active and inactive isoforms, with the latter potentially antagonizing antitumor effects [27,43]. Thus, the balance between pro-tumorigenic and antitumor isoforms may determine clinical outcomes and influence therapeutic strategies targeting GSDMB.

This study provides a new perspective on anti-tumor therapy by modulating GSDMB-mediated pyroptosis. Accumulating evidence showed modulating the apoptosis-pyroptosis transition can empower cancer therapy. For example, GSDMB^iso3^ overexpression alone did not alter tumor growth in colon cancer xenograft models, but PD-1 blockade enhanced pyroptosis and antitumor immunity by reactivating exhausted CD8^+^ T cells [13]. Delivery of the DNA methyltransferase inhibitor decitabine elevates GSDME expression, shifting cell death from apoptosis to pyroptosis, while combined with p53 and immune checkpoint modulation, this strategy effectively kills apoptosis-resistant tumor cells via caspase3/GSDME axis and promotes T cell infiltration and activation, thereby enhancing anti-PD-L1 therapy and the systemic antitumor immune response [49]. The activation of NF-κB by Poly(ADP-ribose) polymerase inhibitors (PARPis) contributes to the secretion of TNF-ɑ and subsequent pyroptosis via TNFR1-caspase 8-GSDMD/E axis, and thereby enhances tumor-targeting immune responses [50]. In this study, we provided evidence to support the hypothesis that miR-BART12-3p inhibitor can switch apoptosis to pyroptosis in EBV-positive GC cancer. Compared to other pyroptosis inducers, miR-BART12-3p inhibitor specifically targets EBV-positive tumor cells, which may substantially restrict pyroptosis to the tumor site and reduce the risk of damage to normal tissues. Given the National Comprehensive Cancer Network (NCCN) guidelines’ recommendation of PD-1/PD-L1 immunotherapy for EBVaGC, we propose that combining miR-BART12-3p inhibitor with PD-1 blockade represents a novel therapeutic avenue for enhancing anti-tumor efficacy in EBVaGC. CAR-T and CAR-NK technologies were increasingly used in cancer therapy, and miR-BART12-3p inhibitor might also empower CAR-T and CAR-NK technology in the treatment of EBVaGC. In addition to the mechanism proposed in our study, other EBV-encoded factors may also play important roles. Specifically, the viral IL-10 homolog (vIL-10), encoded by BCRF1, inhibits NK cell-mediated lysis of EBV-positive lymphoma cells [51]. Moreover, latent membrane protein 2A (LMP2A) activates PI3K/AKT signaling, leading to upregulation of coagulation factor F3 and promoting platelet aggregation around NK cells, thereby impairing their cytotoxicity against EBVaGC [52]. These findings, together with our results, suggest that the immune-resistant phenotype observed in EBVaGC likely results from a combination of viral factors, and further studies are warranted to delineate the relative contributions of each mechanism.

Our study elucidates a novel miRNA-mediated immune evasion strategy employed by EBV and highlights potential therapeutic targets for EBVaGC. Several limitations of this study should be acknowledged. The focus on limited EBV-positive GC cell lines may not fully reflect the heterogeneity of gastric cancers (due to the heterogeneity of cleavable GSDMB isoform expression levels). As a proof of concept, our xenograft model proved that inhibition of miR-BART12-3p can sensitize EBV-positive GC cells to killer lymphocyte-mediated pyroptosis, however, it did not mimic the tumor immune microenvironment in GC patients. Due to the lack of isoform-specific GSDMB data in TCGA-STAD, we were unable to directly assess the correlation between miR-BART12-3p and the specific GSDMB isoform (GSDMB isoform 3) in our study. A large cohort of EBV-positive gastric cancer patients is needed to further validate the correlation between the miR-BART12-3p/GSDMB axis and clinicopathological features as well as survival prognosis. We acknowledge that further validation using BAC mutagenesis or CRISPR editing to knock out miR-BART12-3p in the EBV genome would further strengthen our findings. Unfortunately, we were unable to establish a miR-BART12-3p deletion mutant using the BAC-M81. An alternative approach would have been to use AGS/B95.8 cells, given that B95.8 virus lacks expression of most BARTs, including miR-BART12-3p. However, we were technically unable to pursue this strategy, as we have no access to the B95.8 virus at this point. Despite these limitations, the existing data from alternative approaches in our study provide consistent and compelling support for our conclusions.

In summary, this study demonstrated the EBV-encoded miR-BART12-3p facilitates immune escape by suppressing GSDMB-dependent pyroptosis, which enhances the understanding of viral strategy to evade immune surveillance and provides a potential molecular target for developing novel immunotherapeutic strategies against EBV‑positive gastric cancer.

## Materials and methods

### Ethics statement

Work with human blood was approved by Ethics Committee of Medical Research of Cancer Hospital of Shantou University Medical College (2026-KY-022), and written informed consents were obtained from all adult blood donors. The animal experiments were reviewed and approved by the Ethics Committee of Animal Experiments of Shantou University Medical College (SUMCXM2025-019).

### Cell culture and reagents

AGS (CRL-1739, ATCC) and KATO3 (SCSP-573, Cell Bank, Chinese Academy of Sciences) are EBV-negative GC cell lines. The EBV-positive GC cell line AGS-EBV was established by infecting AGS cells with recombinant EBV strain M81, as previously described [40]. SNU719 (provided by Dr. Myung-SooKang at Sungkyunkwan University School of Medicine, South Korea) and YCCEL1 (provided by professor Qian Tao at the University of Hong Kong) are patients-derived natural EBV-positive GC cell lines. All GC cell lines were routinely cultured in RPMI 1640 medium (#E600028-0500, Sangon Biotech, Shanghai) supplemented with 10% fetal bovine serum (FBS) (#S711-001S, Lonsera, Uruguay) and 100 mg/ml penicillin-streptomycin solution (#BL505A, Biosharp). NK-92MI cells were provided by professor Feng Shao (Beijing Institute of Life Sciences) and cultured in NK-92MI cell culture medium (#CM-0533, Pricella, Wuhan). All cell cultures were maintained at 37°C in a humidified 5% CO_2_ incubator. All synthetic oligonucleotides including miR-BART12-3p inhibitor and mimic (listed in S2 Table) were purchased from RiboBio Technologies (Guangzhou, China), and transfections were conducted using RNAiMAX (#13778150, Invitrogen) according to the manufacturer’s instructions.

### Antibodies

Primary antibodies used in this study: rabbit anti-GSDMB (1:1000, #ab215729, Abcam), mouse anti-GSDMD (1:1000, #sc-81868, Santa Cruz), rabbit anti-cleaved GSDMD (1:1000, #10137S, CST), rabbit anti-GSDME (1:1000, #ab215191, Abcam), rabbit anti-granzyme A (1:1000, #ab209205, Abcam), HRP-conjugated beta-actin monoclonal antibody (1: 5000, #HRP-60008, Proteintech), rabbit anti-PARP1 (1:1000, #80174-1-RR, Proteintech), rabbit anti-caspase 3/P17/P19 (1:1000, #19677-1-AP, Proteintech). Secondary antibodies included horseradish peroxidase (HRP)-conjugated goat anti-mouse antibody (1:5000, #7076S, CST) or HRP-conjugated goat anti-rabbit antibody (1:5000, #7074S, CST).

### Live-cell imaging of co-culturing NK-92MI cells and GC cells

To observe the killing kinetics of GC cells by NK cells, we monitored the co-culture of NK cells and GC cells using a live-cell imaging device (DeltaVision Elite Live Cell Station, GE). We seeded 2 × 10^5 GC cells in 20 mm glass-bottom cell culture dishes (#801001, NEST) coated with poly-L-lysine (#PB180523, Pricella, Wuhan). After 12 h, 1 mL of serum-free medium containing 4 μg/mL calcein AM (#C2012, Beyotime, Shanghai) was added to the GC cells, followed by incubation in an incubator at 37°C for 30–45 min. After removing the supernatant from the culture dish and washing the cells twice with 1 × PBS, NK-92MI cells was added to the culture dish, with an effector-to-target ratio (E/T) of 4. To accommodate the killing kinetics of NK-92MI cells against different GC cells, the live-imaging duration was set to 8–12 hours, with acquisition every 5 minutes. Following live-cell time-lapse recording, all cells in each recorded video were quantified based on the morphological characteristics by at least two researchers in a blinded manner. In the microscopic images of GC cells co-cultured with NK-92MI cells, cells displaying cell swelling and membrane ballooning were defined as pyroptotic cells, whereas dying cells without obvious bubble formation or presenting only membrane blebbing, as well as surviving cells, were classified as non-pyroptotic cell death. The percentage of pyroptotic cells was calculated and presented as the mean ± standard deviation (SD) based on five videos from three independent biological experiments.

### Cell viability and cytotoxicity assays

Cell viability of gastric cancer cells was determined with a Cell Counting Kit-8 (CCK-8; #A311-01/02, Vazyme, Nanjing). Cytotoxicity of cytotoxic lymphocytes was measured by the Lactate Dehydrogenase (LDH) release assay using an LDH Cytotoxicity Assay Kit (#C0017, Beyotime, Shanghai) according to the manufacturer’s instructions. For cell viability assay, all GC cells used for comparison were divided into four groups: (1) medium without cells, (2) medium containing only GC cells, (3) medium containing only NK-92MI cells or activated CD8^+^ T cells, and (4) medium containing both GC cells and cytotoxic lymphocytes. GC cells were seeded into a 96-well microplate at a number of 3 × 10^4 cells/well and allowed to adhere to the plate for 12 h. The culture medium of Groups 1 and 2 was replaced with 100 μL fresh medium, while that of Groups 3 and 4 was replaced with 100 μL medium containing NK-92MI cells at an E/T ratio of 4, or activated CD8^+^ T cells at an E/T ratio of 10. This was followed by a further 3-hour incubation at 37 °C. Then, 10 µL of CCK-8 reagent was added to each well, and after incubation for 1 h at 37°C, optical densities (OD) of stained cells were measured at 450 nm using a Spark multimode microplate reader (Tecan). The OD value of Group 1 was first subtracted from each group OD value, and then the 4-hour cell viability (%) was calculated using the following formula: (Group 4 - Group 3)/ Group 2 × 100. Results are presented as mean ± SD of three independent experiments. For LDH release assay, four groups were set up to measure OD values at 490 nm in triplicate wells in 96-well plates. The four groups are listed as follows: (1) culture medium background control, (2) target cells (T), (3) effector cells (E), (4) mixed wells with an E/T ratio of 4 for NK cells or 10 for activated CD8^+^ T cells, and (5) maximum LDH release positive control. After subtracting the background absorbance (Group 1) from all groups, the percentage of LDH release (cytotoxicity) at 4 hours was calculated using the formula: (Group 4 - Group 3 - Group 2) / (Group 5 - Group 2) × 100. Results are presented as mean ± SD of three independent experiments.

### Western blotting

Cells were harvested and lysed in RIPA lysis buffer (#R0020, Solarbio, Beijing) supplemented with protease and phosphatase inhibitors (#P1045, Beyotime, Shanghai). Protein concentrations of cell lysates were quantified with a BCA Protein Assay Kit (#G3522-2500T, GBCBIO, Guangzhou). Then, 20 µg protein of each sample was separated by 10% sodium dodecyl sulfate-poly-acrylamide gel electrophoresis (SDS-PAGE) and transferred onto a PVDF membrane (#IPVH00010, Millipore, USA). After blocking with 5% skim milk, membranes were incubated with primary antibodies at 4 °C overnight, followed by incubation with secondary antibodies at room temperature for an hour. Visualization was carried out using an enhanced chemiluminescence (ECL) system (#E412-02, Vazyme, Nanjing), and pictures were captured with an Amersham Imager 600RGB (GE Health).

### Isolation of primary human NK cells and CD8^+^ T cells

Human peripheral blood mononuclear cells (PBMCs) were separated from the peripheral blood of healthy volunteers using Ficoll-Paque PLUS cell separation solution (#17144002, Cytiva). Pure and untouched primary human NK cells were negatively isolated from PBMCs using the Dynabeads Untouched Human NK Cells Kit (#113-49D, Invitrogen) according to the manufacturer’s instructions. Purity of isolated NK cells (defined as CD3-CD56+) was assessed by flow cytometry with a NovoCyte Flow Cytometer (ACEA Biosciences) using FITC Anti-Human CD3 (#35-0038, Tonbo Biosciences) and APC Anti-Human CD56 (NCAM) (MY31) (#20-0564, Tonbo Biosciences). Isolated primary NK cells were cultured in RPMI 1640 medium supplemented with 10% FBS, 1% penicillin-streptomycin, and 15ng/mL human IL-2 Recombinant Protein (#200-02-100UG, PeproTech) for 24–48 hours before use in functional assays. AGS and AGS-EBV cells were co-cultured with primary human NK cells from three independent donors at an effector-to-target (E:T) ratio of 20:1. CD8^+^ T cells were purified from PBMCs with MojoSort Human CD8 T cell Isolation Kit (#480129, BioLegend) according to the manufacturer’s instructions. Cells were stained with FITC Anti-Human CD3 (#35-0038, Tonbo Biosciences) and APC Anti-Human CD8a (#20-0089, Tonbo Biosciences), and analyzed by flow cytometry. Isolated CD8^+^ T cells were also cultured in RPMI 1640 medium supplemented with 10% FBS, 1% penicillin-streptomycin, and 15ng/mL human IL-2 Recombinant Protein. To activate the isolated CD8^+^ T cells, cells were co-stimulated with anti-human CD3 Antibody (#300302, BioLegend) and anti-human CD28 Antibody (#377804, BioLegend). Transcript levels of activated markers (CD25, IFNG, GZMA, GZMB) were quantified via RT-qPCR. Finally, the activated cytotoxic CD8^+^ T cells were co-cultured with AGS or AGS-EBV gastric cancer cells at an effector/target cell (E/T) ratio of 10.

### RT-qPCR and stem-loop RT-qPCR

The relative expression of mRNA and miRNA was examined by RT-qPCR and stem-loop RT-qPCR, respectively. Briefly, total RNA was extracted using TRIzol reagent (#9108/9109, Takara) according to the manufacturer’s instructions. For RT-qPCR analysis, mRNAs were reverse transcribed using HiScript III RT SuperMix (#R323, Vazyme, Nanjing) and the cDNA was quantified by qPCR with ChamQ SYBR qPCR Master Mix (#Q311, Vazyme, Nanjing) in triplicate. For all analyses, *ACTB* (β-actin) served as the internal control, and the expression of target genes was normalized to control samples. For stem-loop RT-qPCR, total RNA preparations were reverse-transcribed with a miRNA 1^st^ Strand cDNA Synthesis Kit (#MR101-01/02, Vazyme, Nanjing), and then qPCR was performed as described above. For miRNA analysis, *RNU6B* served as the internal control and the expression of miRNAs was normalized to control samples. GSDMB-specific qPCR primers are listed in Table 1, and other primers used are listed in S1 Table.

### Establishment of a GSDMB^iso3^-expressing vector

To generate a GSDMB^iso3^-expressing vector, total mRNA extracted from AGS-EBV and SNU719 cells was reversely transcribed into cDNA. The GSDMB cDNA was amplified with PCR and cloned into pcDNA3.1-3×Flag vector. All primers used are listed in S1 Table. GSDMB^iso3^-expressing plasmid was verified by sanger sequencing and large-scale preparation was conducted with a EndoFree Maxi Plasmid Kit (#DP117, TIANGEN, Beijing) for subsequent experiments.

### Luciferase reporter assays

The full-length 3’-UTR of GSDMB was cloned into the pGL3 luciferase reporter vector between the XbaI and FseI sites, using a ClonExpress Ⅱ One Step Cloning Kit (#C112, Vazyme, Nanjing). Mutations of the GSDMB 3’-UTR were then generated in the complementary sites that bind to the seed regions of miR-BART12-3p by site-directed mutagenesis with a ClonExpress MultiS One Step Cloning Kit (#C113, Vazyme, Nanjing). PCR primers are listed in S1 Table. Wild-type or mutant 3’-UTR luciferase reporter vectors were co-transfected with miR-BART12-3p mimic or control into AGS cells, or co-transfected with miR-BART12-3p inhibitor or control into AGS-EBV cells. Luciferase activity was measured 48 h after transfection using a Dual-Lumi II Luciferase Assay Kit (#RG089M, Beyotime, Shanghai) on a Spark multimode microplate reader (Tecan). All assays were performed in triplicate. The oligonucleotide sequences are listed in S2 Table.

### Tumor xenograft mouse model

To assess the role of EBV-miR-BART12-3p in regulating NK cell-mediated killing effects of EBV-positive GC xenografts, 6-week-old female NVG-hIL15 mice (#GM-NVG-210001, Model Organisms, Shanghai, China) were used. This mouse model was generated by introducing a humanized IL15 gene via homologous recombination into the severely immunodeficient NSG mouse background (which lacks T, B, and NK cells). This modification aims to support the subsequent proliferation and function of transplanted human NK cells. Mice were subcutaneously transplanted with two EBV-positive GC cells (AGS-EBV and SNU719) transfected with miR-BART12-3p inhibitor (In-B12-3p) or its corresponding negative control inhibitor (In-NC). Each mouse received 5 × 10^6 cells (AGS-EBV or SNU719), with 4 mice per group across 4 experimental groups. Tumor volumes were measured regularly. When tumors reached 100–200 mm^3^, mice were randomly allocated to receive either an intratumoral injection of NK cells or vehicle controls (normal saline). The experiments were terminated 7 days after NK cell injection. Tumor volumes were calculated using the formula (A × B^2^/2), where A is the largest diameter and B is the perpendicular diameter. At the indicated time points, mice were euthanized, and tumors were collected and weighed.

### Flow Cytometric Analysis of Cell Death

Annexin V/7-AAD staining was performed to characterize NK cell-induced cell death in gastric cancer cells. Briefly, AGS and AGS-EBV cells were seeded into six-well plates and cultured overnight. The cells were pre-stained with 4 μg/mL calcein AM (#C2012, Beyotime, Shanghai) at 37°C for 30 min, followed by two washes with PBS to eliminate residual unbound dye. Labeled target cells were subsequently co-cultured with NK-92MI effector cells at an effector-to-target (E:T) ratio of 4:1 for 0, 2, or 4 hours. At each time point, co-cultured cells were harvested, washed twice with ice-cold PBS, and resuspended in 1 × Binding Buffer (#abs50008-100T, Absin, Shanghai). Cells were then stained with APC-conjugated Annexin V and 7-AAD according to the manufacturer’s instructions, and incubated at room temperature in the dark for 5 minutes. After staining, samples were immediately analyzed by flow cytometry with a NovoCyte Flow Cytometer (ACEA Biosciences), with calcein-AM fluorescence used to gate on target gastric cancer cells and exclude NK cells. At least 100,000 events within the calcein-positive gate were acquired per sample. Data were analyzed using FlowJo software. The percentages of early apoptotic cells (Annexin V^+^/7-AAD^-^), late apoptotic/necrotic/pyroptotic cells (Annexin V^+^/7-AAD^+^), and total Annexin V-positive cells were quantified within the target cell population.

### Statistical analysis

Data are shown as mean ± SD (unless otherwise specified) of at least three independent experiments. Statistical analyses were performed with IBM SPSS Statistics 26 software. Differences were considered to be statistically significant at values of *P* < 0.05 by Student’s t-test for two groups, and one-way ANOVA (analysis of variance) analysis for multiple groups. *P < 0.05, **P < 0.01, and ***P < 0.001. In our study, one-way ANOVA was followed by specific multiple-comparison tests, among which Dunnett's test was applied to the cell experiments that compared three siGSDMB constructs against a single negative control, as this constitutes the standard approach for treatment-versus-control contrasts. Tukey's honestly significant difference (HSD) test was applied to end-point tumour weights among four xenograft groups to assess all pairwise comparisons. Tumour growth curves were analyzed using two-way repeated-measures ANOVA with Geisser-Greenhouse correction, followed by Šídák's multiple-comparison test at each time point.

## Supporting information

S1 Movie Live cell imaging of AGS cells co-culture with NK cell. (MP4)

S2 Movie Live cell imaging of AGS-EBV cells co-culture with NK cells. (MP4)

S3 Movie Live cell imaging of KATO3 cells co-culture with NK cells. (MP4)

S4 Movie Live cell imaging of SNU719 cells co-culture with NK cells. (MP4)

S5 Movie Live cell imaging of AGS-EBV cells transfected with empty vector followed by co-culture with NK cells. (MP4)

S6 Movie Live cell imaging of AGS-EBV cells transfected with GSDMB^iso3^ over-expression vector followed by co-culture with NK cells. (MP4)

S7 Movie Live cell imaging of SNU719 cells transfected with empty vector followed by co-culture with NK cells. (MP4)

S8 Movie Live cell imaging of SNU719 cells transfected with GSDMB^iso3^ over-expression vector followed by co-culture with NK cells. (MP4)

S9 Movie Live cell imaging of AGS cells transfected with negative control mimic followed by co-culture with NK cells. (MP4)

S10 Movie Live cell imaging of AGS cells transfected with miR-BART12-3p mimic followed by co-culture with NK cells. (MP4)

S11 Movie Live cell imaging of AGS-EBV cells transfected with negative control inhibitor followed by co-culture with NK cells. (MP4)

S12 Movie Live cell imaging of AGS-EBV cells transfected with miR-BART12-3p inhibitor followed by co-culture with NK cells. (MP4)

S13 Movie Live cell imaging of SNU719 cells transfected with negative control inhibitor followed by co-culture with NK cells. (MP4)

S14 Movie Live cell imaging of SNU719 cells transfected with miR-BART12-3p inhibitor followed by co-culture with NK cells. (MP4)

S1 Fig EBV-positive GC cells escaping from NK cell-mediated pyroptosis is correlated with the downregulation of GSDMB. Western blot analysis of caspase 3 and PARP cleavage in AGS and AGS-EBV cells after 6-hour co-culture with NK-92MI cells. β-Actin served as a loading control.
(DOCX)

S2 Fig EBV-positive GC cells escaping from primary human NK cell-mediated pyroptosis is correlated with the downregulation of GSDMB. (A) Representative flow cytometry dot plots showing the gating strategy for CD3^-^CD56^+^ NK cells before and after isolation from PBMCs of three independent donors (#1, #2, #3). Quantification of NK cell purity is shown on the right (n = 3). (B) Cell viability of AGS and AGS-EBV cells after 4-hour co-culture with primary NK cells from three independent donors (E:T ratio = 20:1), measured by CCK-8 assay (n = 3). (C) LDH release assay showing lytic cell death in AGS versus AGS-EBV cells after 4-hour co-culture with primary NK cells from the same donors (n = 3). (D) Western blot analysis of GZMA and GSDMB cleavage in AGS and AGS-EBV cells after 6-hour co-culture with primary NK cells from three donors. Full-length (FL, 46 kDa) and cleaved N-terminal (N-, ~ 30 kDa) GSDMB are indicated. β-Actin served as a loading control. Data represent mean ± SD. Statistical significance was determined by Student’s t-test (*P < 0.05, **P < 0.01, ***P *<* 0.001; *ns*, not significant).
(DOCX)

S3 Fig EBV-encoded miR-BART12-3p directly targets the 3’-UTR of GSDMB transcripts to down-regulate the expression of GSDMB. HEK293T cells were co-transfected with a plasmid expressing GSDMB isoform 3 and the miR-BART12-3p mimic or their corresponding negative controls, and then (A) the expression of miR-BART12-3p was detected using stem-loop qRT-PCR and (B) the protein level of GSDMB was determined by western blot analysis after 48 hours.
(DOCX)

S4 Fig miR-BART12-3p inhibitor promotes NK cell-mediated GSDMB cleavage *in vivo* and controls EBV-positive gastric cancer growth. (A, B) Stem-loop qRT-PCR detection of miR-BART12-3p expression in harvested xenograft tissues from AGS-EBV **(A)** and SNU719 **(B)** tumor-bearing mice at the experimental endpoint. Tumors were derived from cells transfected with miR-BART12-3p inhibitor (In-B12-3p) or negative control inhibitor (In-NC). Data are presented as mean ± SD; *P < 0.05; *ns*, no significant difference.
(DOCX)

S1 Table Sequence of PCR and qPCR. (DOCX)

S2 Table Synthetic oligonucleotide sequences used in this article. (DOCX)
