# Supplementary material for: Downregulation of GSDMB by EBV-encoded miR-BART12-3p suppresses killer lymphocyte-mediated pyroptosis in gastric cancer cells
Source: PLoS Pathog. 2026 Aug 5;22(8):e1014495. doi: 10.1371/journal.ppat.1014495 (PMC13466057; doi:10.1371/journal.ppat.1014495)
Supplement: Supplementary file 1 [file ppat.1014495.s015.docx]

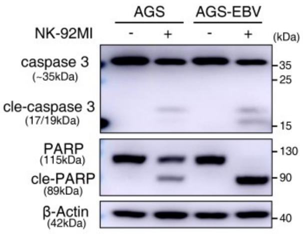

AGS
AGS-EBV
NK-92MI
-
+
-
+
(kDa)
caspase 3
(~35kDa)
cle-caspase 3
(17/19kDa)
PARP
(115kDa)
cle-PARP
(89kDa)
β-Actin
(42kDa)
35
25
15
130
90
40
