# Supplementary material for: Downregulation of GSDMB by EBV-encoded miR-BART12-3p suppresses killer lymphocyte-mediated pyroptosis in gastric cancer cells
Source: PLoS Pathog. 2026 Aug 5;22(8):e1014495. doi: 10.1371/journal.ppat.1014495 (PMC13466057; doi:10.1371/journal.ppat.1014495)
Supplement: Supplementary file 2 [file ppat.1014495.s016.docx]

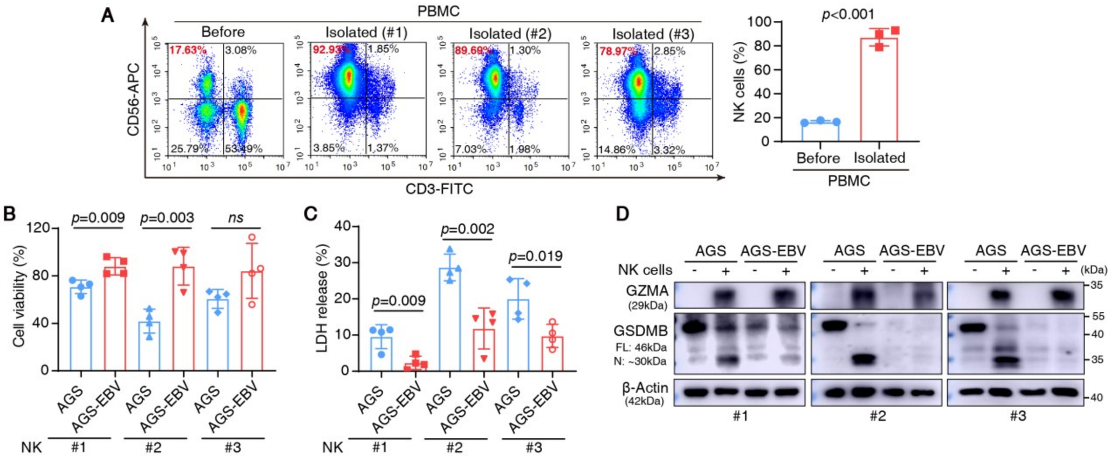
A
PBMC
Before
Isolated (#1)
Isolated (#2)
Isolated (#3)
CD56-APC
CD3-FITC
17.63%
3.08%
25.79%
53.49%
92.93%
1.85%
3.85%
1.37%
89.69%
1.30%
7.03%
1.98%
78.97%
2.85%
14.86%
3.32%
p<0.001
NK cells (%)
Before
Isolated
PBMC
B
Cell viability (%)
p=0.009
p=0.003
ns
AGS
AGS-EBV
NK
#1
#2
#3
C
LDH release (%)
p=0.009
p=0.002
p=0.019
D
AGS
AGS-EBV
NK cells
(kDa)
GZMA
(29kDa)
GSDMB
FL: 46kDa
N: ~30kDa
β-Actin
(42kDa)
#1
#2
#3
