# Supplementary material for: Downregulation of GSDMB by EBV-encoded miR-BART12-3p suppresses killer lymphocyte-mediated pyroptosis in gastric cancer cells
Source: PLoS Pathog. 2026 Aug 5;22(8):e1014495. doi: 10.1371/journal.ppat.1014495 (PMC13466057; doi:10.1371/journal.ppat.1014495)
Supplement: Supplementary file 3 [file ppat.1014495.s017.docx]

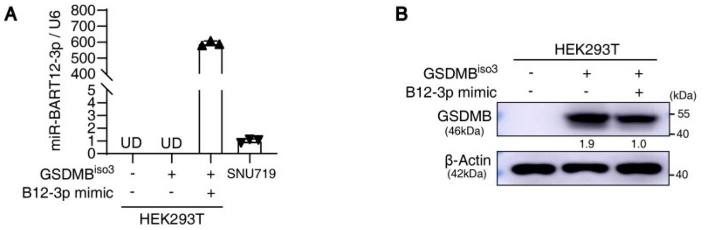
A
miR-BART12-3p / U6
800
700
600
500
400
5
4
3
2
1
0
UD
UD
GSDMB^iso3
B12-3p mimic
SNU719
HEK293T
B
HEK293T
GSDMB^iso3
B12-3p mimic
(kDa)
GSDMB
(46kDa)
55
40
1.9
1.0
β-Actin
(42kDa)
40
