# Supplementary material for: Downregulation of GSDMB by EBV-encoded miR-BART12-3p suppresses killer lymphocyte-mediated pyroptosis in gastric cancer cells
Source: PLoS Pathog. 2026 Aug 5;22(8):e1014495. doi: 10.1371/journal.ppat.1014495 (PMC13466057; doi:10.1371/journal.ppat.1014495)
Supplement: Supplementary file 4 [file ppat.1014495.s018.docx]

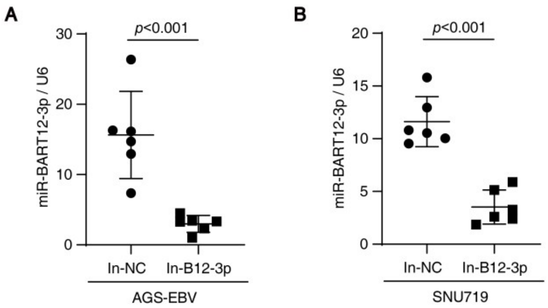

A
p<0.001
miR-BART12-3p / U6
30
20
10
0
In-NC
In-B12-3p
AGS-EBV
B
p<0.001
miR-BART12-3p / U6
20
15
10
5
0
In-NC
In-B12-3p
SNU719
