# Supplementary material for: Downregulation of GSDMB by EBV-encoded miR-BART12-3p suppresses killer lymphocyte-mediated pyroptosis in gastric cancer cells
Source: PLoS Pathog. 2026 Aug 5;22(8):e1014495. doi: 10.1371/journal.ppat.1014495 (PMC13466057; doi:10.1371/journal.ppat.1014495)
Supplement: Supplementary file 5 [file ppat.1014495.s019.docx]

**S1 Table. Sequence of PCR and qPCR**

| Name | Sense（5'-3'） |
| --- | --- |
| BART12-3p (F) | GCGTCCTGTGGTGTTTGGT |
| BART12-3p (R) | AGTGCAGGGTCCGAGGTATT |
| BART12-3p (RT) | GTCGTATCCAGTGCAGGGTCCGAGGTATTCGCACTGGATACGACAACCAC |
| BART20-3p (F) | CGCATGAAGGCACAGCCT |
| BART20-3p (R) | AGTGCAGGGTCCGAGGTATT |
| BART20-3p (RT) | GTCGTATCCAGTGCAGGGTCCGAGGTATTCGCACTGGATACGACGGTAAC |
| U6 (F) | CTCGCTTCGGCAGCACA |
| U6 (R) | AACGCTTCACGAATTTGCG |
| U6 (RT) | AACGCTTCACGAATTTGCG |
| GSDMB-PCR (F) | CTTGGTACCGAGCTCGGATCCATGTTCAGCGTATTTGAGGAAATCA |
| GSDMB-PCR (R) | GTAGTCGAAGGGCCCTCTAGAGGAAGAGACAGAGGTAGGCCCC |
| Wt-GSDMB-3'-UTR (F) | AGATCGCCGTGTAATTCTAGACTACAAAAGCCCTTTCTCCCCA |
| Wt-GSDMB-3'-UTR (R) | ATGTCTGCTCGAAGCGGCCGGCCTTAACATGCACAACCTGCCACT |
| Mut-GSDMB-3'-UTR (F) | ATCCTGTACAACGTAACACCTCTTTAAAACAAGCAGC |
| Mut-GSDMB-3'-UTR (R) | AAGAGGTTGTACGTTGTACAGGATGGTAGTGGCGATG |
| CD25 (F) | CGCAGAATAAAAAGCGGGTCA |
| CD25 (R) | ACTTGTTTCGTTGTGTTCCGA |
| IFNG (F) | TCGGTAACTGACTTGAATGTCCA |
| IFNG (R) | TCGCTTCCCTGTTTTAGCTGC |
| GZMA (F) | TCTCTCTCAGTTGTCGTTTCTCT |
| GZMA (R) | GCAGTCAACACCCAGTCTTTTG |
| GZMB (F) | TACCATTGAGTTGTGCGTGGG |
| GZMB (R) | GCCATTGTTTCGTCCATAGGAGA |
| β-actin (F) | TCCTCCTGAGCGCAAGTACTC |
| β-actin (R) | CGGACTCGTCATACTCCTGCTT |
