# Supplementary material for: Downregulation of GSDMB by EBV-encoded miR-BART12-3p suppresses killer lymphocyte-mediated pyroptosis in gastric cancer cells
Source: PLoS Pathog. 2026 Aug 5;22(8):e1014495. doi: 10.1371/journal.ppat.1014495 (PMC13466057; doi:10.1371/journal.ppat.1014495)
Supplement: Supplementary file 6 [file ppat.1014495.s020.docx]

**S2 Table. Synthetic oligonucleotide sequences used in this article**

| Name | Sense（5'-3'） |
| --- | --- |
| EBV-miR-BART12-3p mimic | 5’ UCCUGUGGUGUUUGGUGUGGUU 3’  3’ AGGACACCACAAACCACACCAA 5’ |
| Mimic NC | 5’ UUUGUACUACACAAAAGUACUG 3’  3’ AAACAUGAUGUGUUUUCAUGAC 5’ |
| EBV-miR-BART12-3p inhibitor | AACCACACCAAACACCACAGGA |
| Inhibitor NC | CAGUACUUUUGUGUAGUACAAA |
| siGSDMB#1 | GCAGGAUCUAGAGCAAAGATT |
| siGSDMB#2 | GCAGGAUCUAGAGCAAAGATT |
| siGSDMB#3 | CAGCCAACCAUUCUUUGAUTT |
| siGSDMB Cont. | UUCUCCGAACGAGUCACGUTT |
